# The Three Powerful Traditions of Thinking that Constitute Homeopathy *

**DOI:** 10.1055/s-0043-1775814

**Published:** 2024-01-29

**Authors:** Josef M. Schmidt

**Affiliations:** 1 Institute of Ethics, History, and Theory of Medicine, Ludwig Maximilian's University, Munich, Germany

**Keywords:** history of medicine, theory of medicine, history of knowledge, homeopathy, philosophy

## Abstract

By means of a historical, classical philological and philosophical approach, this paper attempts to demonstrate that homeopathy is based on three powerful traditions of thinking, which can be traced back to Ancient Greece's pre-Socratic era. Actually, it seems to be constituted by what may be termed *lógos* -, *hómoion* - and *iásthai* -thinking: that is, thinking in terms of rationality, similarity and healing. By contrast, modern medicine tends to be aligned with just one of these traditions, at the expense of the others, this being not without risk and adverse effects. It is mainly determined by the first type of rationality that genealogically derives from, and is therefore compatible with, the logic of economics whose predominance in the health care systems of modern societies is progressively rising. Homeopathy, however, may not be sufficiently and fairly understood without taking into account the complementary forms of thinking on which it also rests, such as the principle of similarity in an all-encompassing sense, and ancient healing knowledge in the tradition of catharsis. As a corollary of being essentially constituted by the three, homeopathy may persistently be in need of a dynamic equilibrium of its three constituent bases. Attempts to approach homeopathy from only one of the indicated modes of thinking fail to grasp its essence and result in figments or caricatures of what homeopathy was originally meant to be.

## Introduction

On its long and rocky way from the 18th to the 21st century, homeopathy has been facing and overcoming many obstacles, ideologically, institutionally and financially, to finally find a reasonable place within the health care systems of many countries of the world. Its biggest threat, however, has been taking shape only unobtrusively in recent decades, still appearing to be invisible to most of its advocates as well as critics. Certainly, nowadays everybody takes notice of the rapid socioeconomic, political and scientific changes in modern societies, ranging from secularisation, rationalisation and globalisation to social networking, artificial intelligence and genetic engineering. However, to also recognise what, simultaneously to these alleged advancements, is getting lost in terms of human culture, one has to take a step back into history and become aware of what dimensions of life and thought, for example in the case of homeopathy, its founder Samuel Hahnemann still had access to.

The main thesis of this paper is that homeopathy is fundamentally based on three powerful traditions of thinking, all of which originated in Ancient Greece in the pre-Socratic era, from which, however, in modern societies, one ( *lógos-* thinking) has achieved more and more supremacy, if not exclusivity, at the expense of the other two ( *hómoion* - and *iásthai-* thinking). (As will be shown later, the term *lógos* refers to a kind of impersonal rationality characteristic of monetarised societies, *hómoion* to thinking in analogies and connections by similarity, *iásthai* to a way of thinking crucial for the practical art of healing). While homeopathy has been founded and constituted at a time when educated doctors and patients were still conversant with a broad range of ancient traditions, today's reduction of scientific rationality on quantifiability, reproducibility, standardisability, etc., fails to grasp the essence of homeopathy, thus academically attacking or defending nothing but an anaemic caricature of what homeopathy was originally meant to be.

To understand, practice and promote homeopathy, even today, in its genuine sense and cultural context, and in fact prevent it from irrelevance and oblivion, a detailed comprehension of its intellectual roots is needed, more than ever before. Ultimately, the new categories suggested here on the basis of a serious philological, historical and philosophical study may also prove to be helpful to come to terms with many other basic universal and existential problems of our age.

## 1. *Lógos* -thinking

The form of thinking that today clearly predominates the world, with all its risks and adverse effects, may be traced back to the 6th century BC, when in Ancient Greece monetarisation took hold. In the following, it will be called *lógos* -thinking.

By the end of the 7th century BC, in the direct neighbourhood of the Greek colonies on the western coast of modern Turkey, in Lydia, the first coins worldwide were minted, initially out of electron, a variable mixture of gold and silver which was mined there in great quantities. The Greeks adopted this invention rapidly and started to mint their own silver coins so that in the 6th century BC wide parts of Magna Grecia, which extended from the Ionic coast to lower Italy and Sicily, consisted of fully monetarised *poleis* (city states). During this particular time, in the 6th century BC, the world saw the emergence of what today we call ‘rational thinking’, in contrast to mythological thinking. This was, *nota bene* , for the first time and unique on the entire globe. 1 2

The new rational view of the world was unprecedented, just as the (now existing) basic functions of money, such as medium of exchange, means of payment, measure of value, store of purchasing power, as well as its general acceptance, exclusivity and sanction by the state. It should be considered that in Homeric times, up to the 7th century BC, the exchange of goods took place entirely without money, just on the basis of personal reciprocity of gifts or communal distribution of sacrificial offerings, yet long-distance trade with the Near East (Egypt and Babylonia) was already carried out to some extent by means of pieces of silver (which, however, due to lack of coinage had to be weighed). The circulation of money coins was thus (so to say) a quantum leap in the intellectual history of humankind.

With minted coins, their exchange or monetary value (i.e., what one gets for them in the marketplace) is no longer identical to their utility value (i.e., the worth of the material) but is determined solely by their coined sign. Thus, it does not rest on its nature ( *phýsis* ) but on convention ( *nómos* ), as Aristotle later, in the 4th century BC, would explain. 3 This difference required and facilitated general abstraction, away from sensually perceptible qualities of the lifeworld toward the idea of an all-pervading purely spiritual value or essence. This act of abstraction, to be performed daily by all market participants, soon seemed to kind of self-evidently belong to the social economic lifeworld. Hence, it must have equally had an impact on the thinking of the first occidental philosophers. Indeed, all properties of the new money can be found there, such as homogeneity, impersonality, universality, boundlessness, abstractness, etc. 4

The first philosophical concepts of the occident that have been handed down to us originated from Miletus, the former leading commercial town on the Ionian coast which in the 6th century BC was first to be entirely monetarised. Here, at that time, Thales, Anaximander and Anaximenes were active. 5

First, Thales put forward the monistic thesis that water ( *hýdor* ) may be the first and only element. 6 For his disciple Anaximander, the eternal *ápeiron* , the undefinable, was the origin of everything, 7 and for his successor Anaximenes, it was the compression or decompression of the immaterial, unlimited and rich air ( *aér* ) that keeps everything together. 8 In light of the wide array of phenomena, the common feature of these first testimonies of rational thinking is the counter-intuitive postulation of an abstract impersonal principle underlying everything—as it were a projection of the monetarisation of the *pólis* onto the *kósmos* .

Xenophanes, born in Colophon, another monetarised polis north of Miletus, after being banned and having moved to Elea, a monetarised polis in Campania (Lower Italy), toward the end of the 6th century BC, propagated one abstract God who—contrary to the anthropomorphic images of gods by Homer and Hesiod—does not resemble humans at all, however, by means of his *noús* (spirit) keeps running everything. 9

Pythagoras was born in Samos, an island off the Ionian coast, but migrated to Kroton, another entirely monetarised Greek polis in Calabria, South Italy, toward the end of the 6th century BC. His disciples, mainly Philólaos in the 5th century BC, taught the counter-intuitive doctrine that the number ( *arithmón* ) is the essence of all things, that numbers are the things themselves, and that even natural things derive from numbers. 10

Heraclitus from Ephesus, again a monetarised polis in Ionia north of Miletus, at the beginning of the 5th century BC, explicitly posed the issue of a mutual turnover of everything against fire—‘just as commodities against gold’. For him, the cosmos was an everlasting living fire that will judge everything. Everything happens according to the *lógos* . The soul has *lógos* that augments itself. Only one thing is wise, to understand the thought ( *epístasthai gnómen* ) which knows to control everything in every way. 11 *Lógos* here means that which holds the world together in its innermost folds, or rather its cognition.

Parmenides probably first studied with Anaximander in Miletus and then went to Elea in Campania (Lower Italy), to study with Xenophanes (the Ionian philosopher who went there before him). There, at the beginning of the 5th century BC, Parmenides demanded: ‘judge according to the *lógos* !’. For him, truth consisted in the fact that thinking ( *noeín* ) and being ( *eínai* ) are the same ( *tó autó* ). Here, being is thought as a whole, as one, imperishable, homogeneous, perfect and aimless. 12 The epistemological division between mental abstraction of the absolute, on the one hand, and the sensory world, on the other, here reaches its peak. Parmenides had an enormous impact on Plato and the entire occidental philosophy.

With Anaxagoras, born in Klazomenai, a monetarised polis in Ionia near to Colophon, after his flight from the Persians, in the 5th century BC, philosophy finally came to Athens. His doctrine that the *noús* has the control over everything, 13 however, was received by the Athenians as adversely as his (and his disciple Archelaos') disciple Socrates' search of ‘the sole right coin’ ( *mónon tó nómisma orthón* ), against which one must change everything else, that is, reasonability ( *phrónesis* ). 14

Until then, pre-Socratic philosophers had consistently been active in authoritarian regimes, such as Miletus, Ephesus, Samos or Colophon, where the new universal power of money was realised in the person of the tyrant vividly and unambiguously. In Athens, which had only become a democracy under Kleisthenes in 508 BC, however, the new *lógos* -thinking faced resistance, particularly as a democracy has to rely on the voluntary cohesion of its citizens which most likely is ensured by common myths, traditions and values rather than by abstract *lógos* -thinking that merely boosts the egoism of every individual. To ward off atheism ( *asébeia* ) and the corruption of the youth, Anaxagoras and Socrates were indeed sentenced to death. 15

Nevertheless, relativisation of convention, accompanying the new *lógos* -thinking, would not be long in coming. As a representative of the new business model of the sophists to teach the new *lógos* for a fee, here just Protagoras (from Abdera in Thrace) may be mentioned who in the 5th century BC taught in Athens that the measure ( *métron* ) of all things is the human ( *ánthropos* ). 16 The absolute separation between being and appearance now was denounced as a construction and given over to the deconstruction and subjective reconstruction of a wealthy new elite.

Eventually, with Plato and Aristotle in the 4th century BC the entire pre-Socratic philosophy was processed and integrated into a classical canonical form which would shape and determine the thinking of the occidental world until today. 17

## 2. *Hómoion* -thinking

Besides this form of money-driven *lógos* -thinking, there always existed another powerful way of thinking, which in the following will be called *hómoion* -thinking.

The fact that similars, kins and kindred things attract each other, are friendly, connect and enjoy each other, while different and extraneous things are hostile and repulse each other, may be familiar and obvious to every archaic human. In fact, the distinction between equal and unequal, own and foreign, and the corresponding social practice of including and excluding is one of the earliest principles of order and orientation of humankind. With the Greeks, family ties and ritual communities have always played an important role so that the word *phíloi* (friends) was originally and primarily related to relatives ( *syggeneís* ). 18

Only with the pre-Socratics (the Greek philosophers that preceded Socrates) the attempt arose to conceptualise the familiar but yet diffuse sense of belonging among congenials as a natural philosophical principle, namely as the *hómoion-homoío* -principle. *Hómoios* (or *homoiótes* , similarity) here means equality of shape and appearance, of form, of feature—that is, a qualitative consubstantiality—while *ísos* indicates a quantitative equality, same degrees of force, power, honour, strength, size or number. *Hómoios* rather is a geometrical and *ísos* an arithmetical equality. The term equal, however, only makes sense as a distinction from and confrontation with its contrary, the notion of inequality. Accordingly, both (complementary) principles, *hómoion-homoío* and *enantíon-enantío* , had to be taken into account to explain all phenomena of nature. While friendship of equals was considered to be the natural course of things, the unity of distinct entities, however, had to be ascribed to a special force. 19

For the Milesian philosophers (Thales, Anaximander, Anaximenes) and the Pythagoreans in Croton, in the 6th century BC, a principle of similarity was still not an issue. The first to touch this thought philosophically was Heraclitus of Ephesus who, in the 5th century BC, declared that the immortal soul after the death of the body ‘turns to its cognate’ ( *prós tó hómogenés* ), probably meaning the primordial fire. 20

Systematic application of the principle of similarity to physics and epistemology only started with Parmenides in Elea in the 5th century BC, in the course of his dialectical handling of the equal and unequal. In the absolute state of being and truth, there is no place for a dynamic *hómoion prós hómoion* . However, the world of opinions consists of opposites which mutually exclude each other, which cannot merge but only mix and thus give rise to the illusory world. To force the heterogeneous elements into a fusion, an all-steering goddess ( *daímon* ) is needed. Only death allows the dissolution of the involuntary *míxis* and reversion to the co-generic. 21

Empedokles of Akragas, a Greek polis in Sicily and (since 572 BC) a democracy, in the 5th century BC conceptualised a more vivacious worldview than Parmenides: his elements yearn for each other, love and enjoy each other, and take part in reasoning. As all-controlling powers, he postulated love ( *philótes* ) and strife ( *neíkos* ). Friendship brings about the connection of all elements, whether similar or dissimilar. Hostility results in separation, but only among dissimilars. Similars cannot antagonise themselves: this would be self-hatred. In the period of *philótes* everything unites in love. Under the rule of *neíkos* the unity breaks apart, between the dissimilars separating strife prevails, and love has to confine itself to friendship of the similar, that is, self-love. This condition, however, signifies the decay of the universe into its elements, each lumping together with themselves. Individual beings can exist only by simultaneous activity of *philótes* and *neíkos* : on the one hand, by strife they are segregated from the cosmic *sphaíros* and, on the other, by love they are held together to the unity of their nature. 22

Anaxagoras from Klazomenai who, as mentioned, had brought Ionian Enlightenment to Athens in the 5th century BC, postulated an infinite number of qualities. They were segregated from the primordial mixture, in which ‘all things were together’ ( *homoú* ), by the *noús* who then was setting the mixture into a vortex-motion ( *perichóresis* ). According to the principle ‘same to same’, thus an increasing differentiation of the secreted subsets was achieved, until de-mixing and mixture, appearing and disappearing were balanced. Cosmogony took place according to the principle ‘related things are brought together’, however not, as with Empedokles, through a movement triggered by desire and craving, but imposed from the outside, by the *noús* , without own involvement. The *noús* arranges everything; but how exactly, Anaxagoras did not explain. 23

While Empedokles and Anaxagoras still assumed a plurality of qualities, the atomists Leukipp and Demokrit, both from Miletus or Abdera, a wealthy and monetarised polis in Thrace, in the 5th century BC postulated a plurality of corpuscles in an empty space, identical in quality and distinguished merely by size and shape. Each of these so-called atoms or *idéai* was thought as uniform, indivisible, imperishable and unchangeable, much like the being ( *eón* ) of Parmenides. What appears as change, origin and decay is just a combination and separation of atoms. The physical monism here, for the first time, is justified by the *hómoion-homoío* -principle: all atoms have to be qualitatively uniform, because only equal can act on equal or be affected by it. On the other side, the principle that equal is led to equal was tried to explain through mechanical causes, such as shaking a sieve with different grain types or stones washed ashore by the surf. Depending on the size and shape of the otherwise equal stones they react to equal mechanical impact in an equal manner so that the small ones will be placed next to the small and the large next to the large. To be sure, this no longer has anything to do with mutual attraction or affinity. Atomists only accept pressure and blow as causes of motion. 24 25

In the Corpus Hippocraticum which originated on the island of Kos, off the southern Ionian coast, in the 5th century BC and which contains writings of different authors and schools, physiological accounts about origin, growth, nutrition and composition of the organism are consistently explained according to the *hómoion-homoío* principle, in the sense of Empedokles, Anaxagoras or Demokrit. To some extent, however, explanations are given also according to the *enantíon-enantío* -principle, which is no contradiction, as friendship of the concurring and hostility of the opposed do not exclude each other. Where opposites exist, for instance between warm and cold or wet and dry, in therapy the contrarium principle ( *tá enantía tón enantíon estín iémata* ) applies. 26 27 28 29 30 The effect of purging substances, however, is explained according to the *hómoion-homoío* principle in the sense that the pharmacon will draw the humour most akin to itself ( *autó katá phýsin málista* ) toward itself, with itself, and excrete it. 31

With the sophists of the 5th century BC, the *hómoion-homoío* -principle was also used as a basis for epistemic relativism, for example, when Protagoras exalted the human being to be the measure of all things, and everybody was supposed to like his/her own, familiar, related and equal ( *tó hómoion tó homoío hedý* ), encouraging a new individualism. 32

## 3. *Iásthai* -thinking

Besides *lógos* -thinking and *hómoion* -thinking, from time immemorial there also was old healing knowledge which does not fit into any of the aforementioned categories and which in the following will be called *iásthai* -thinking—that is, thinking in terms of healing—from the Greek formula: ‘ *ho trósas kai iásetai* ’, which literally means ‘The one having hurt will also cure’. 33

In search of ancient healing knowledge, of course, one may not expect a schoolbook-like definition in the sources but, at most, parables in myths or mnemotechnic verses deducted from many experiences. The earliest reference may seem to be the Cýpria, the epic that describes the prehistory of the Iliad of Homer and originated in the 7th century BC. 34 It contains the history of the Mysian king Telephos who was wounded by Achilles with his spear at the thigh (Mysia was located east of Troas, in the northwest of Asia Minor, at the Sea of Marmara). After the wound (of Telephos) did not heal, the king asked the Lycian oracle of Apollo in Patara (in the southwest of Asia Minor) for advice and got the answer: ‘Only who stroke the wound can heal it’, *ho trósas kai iásetai* . So Telephos moved to the camp of the Greeks, and indeed Achilles cured him.

Homer, in the 7th century BC, reports in the Iliad that Achilles was a pupil of Chiron from whom he had learned the healing art. Chiron was a centaur, half man, half horse, and also in every other respect a divided nature: wild and mild at once, immortal and suffering from an incurable wound. 35 It should be noted that in the oldest reconstructable version of the mythos the way to healing was revealed by the oracle—that is, by Apollo, the god of healing, who in fact merely gave a hint—while the practical application of this hint was performed by the physician Achilles, in finding the right remedy.

Later, the oracle saying was narrowed down rationally and no longer understood as such. As an example, Plinius may be taken, who in the 1st century AD, in his natural history, illustrated the chapter ‘Remedies from rust’ with an explanation that the fabulous healing of Telephos by Achilles was affected by ‘scraping off rust from his sword’. 36 However, such an enormous and paradoxical idea, such as ‘what hurts also heals’ could hardly have arisen from wound treatment. There must have been an underlying general reflection which, for example, is also displayed by the snake on the Aesculapian rod: the wisdom that one can get help out of the threat, that the dangerous and the helpful may be one. Besides primitive defence reactions to hazards, such as killing or eliminating the inimical, a part of humanity may well have recognised early on that utilisation, integration and taming of the threatening may get one further.

Among philosophers, the talk of the thorn or ‘bite’ of philosophy, stimulating the victim to spiritually beneficial counter-reactions, was a common *tópos* , for example with Plato 37 in the 4th century BC or Lukian 38 in the 2nd century AD.

Also in the Jewish–Christian cultural area, the idea of the salubrity of the harmful was known, in the form of a sting, for example, in the book Ecclesiastes 39 which originated in the 3rd century BC or with Paul the Apostle 40 in the 1st century AD.

A generally known form of curative commotion is *kátharsis* , which the classical Greek tragedies (and comedies) were aiming at, and which, through Aristotle in the 4th century BC, was exemplarily defined for the following two thousand years. For him, a tragedy is the imitation of a serious action through which ‘by compassion and fear ( *di‘ eléou kaí phóbou* ) a purification ( *kátharsin* ) of these very affects is brought about’. 41 42 By compassion with the tragic fate of the hero and fear of his hubris which reminds oneself of one's own abysses, the spectators may have the opportunity to transform their own passive suffering into conscious activity and thus overcome it.

As with the ancient Greek proverb *patheín matheín* (learning by suffering), however, this is a rare and great thing that may not be obtained cheaply but requires moral strength and courage. 43

## The Roots of Medicine

These are the three most important ways of thinking or intellectual currents, on which Hahnemann's homeopathy seems to rest and which, apart from that, should be the basis of any kind of medicine that claims to be an art of healing (see below). The different genealogy of the *lógos* -, *hómoion* - and *iásthai* -thinking out of different socio-economical contexts is also mirrored in their development through the epochs of the history of medicine.

1. Thus *lógos* -thinking, which originated from the spirit of monetarised economies in ancient authoritarian regimes, played its largest role in medical systems in which a professionalised medical profession, distinguishing itself from allegedly ignorant laypeople, claimed to know and control the basic processes in the human organism—in perfect analogy to the attitude and conceit of pre-Socratic philosophers thinking to have worked out, by their elitist cognition of the ominous one (be it water, air, fire, number or being), the essence of the cosmos and the processes in society. Examples from medical history range from the rationalistic sections of the Corpus Hippocraticum 44 over Galen and the scholastic medicine to modern high-tech medicine and the reductionist focusing on data, statistics and evidence-based studies.
2. The *hómoion* -thinking, whose conceptualisation within natural philosophy and medicine derives from a natural sensuality and predilection for similars in a more democratic environment, was always strongly represented in medical concepts which put special emphasis on qualities and embeddedness in a meshwork of relations, whether between humans and environment or organs and humours and cosmic elements. The humoral pathology which originally derived from the observation of correspondences, that is, *hómoion* -relations, and from the differentiation of different qualities, may serve here as only half an example, as it soon was superimposed by its other half, its systematisation and dogmatisation by *lógos* -thinking. This appropriation, however, enforced its validity up to the 19th century. Mainly shaped by *hómoion* -thinking, however, was the medicine of Paracelsus 45 who decidedly turned away from scholastic *lógos* -rationality and rather looked for ever new analogies of microcosm and macrocosm, signatures of plants or relationships between celestial bodies, metals and parts of the body, etc., to influence them by alchemistic and magical means. 46
3. The *iásthai* -thinking falls out of an alleged frame of three equivalent principles, insofar as—contrary mainly to the first, a little less to the second—it cannot claim generality, scientificity, quantifiability, standardizability, reproducibility, etc. Because healing by the hurting, in its broad original comprehension, may not be an automatism or natural law, but a high art accessible only to a few adepts and successful probably only in particular cases. From the healing of Telephos by his war-enemy Achilles up to the cathartic purification of the anxiety of the theatre guest, by means of fear for the tragical hero, this type of healing was never a recipe for the masses but would always require from the patient a particular disposition and from the physician a particular skill.

## The Roots of Hahnemann

How did Hahnemann come to incorporate all these three strands of thinking into his conception of homeopathy?

1. As a child of his time, the time of German Enlightenment and Rationalism, Hahnemann's consciousness was largely shaped by *lógos* -thinking. His writings show him, especially until 1810, as a young man who wanted to raise medicine up to the status of a science, preferably mathematical, and find his new art of healing by means of rational theories. 47
2. As a child of his time, however, he also participated in the culture and mood of the Age of German Sentimentalism and later Romantic. 48 This may, on the one hand, explain his pathos considering his morality, idea of man and conception of God, and on the other, his love toward his fellow human beings and their individual peculiarities, that is, the diversity of the qualities of life. This variety of phenomena (and his response, in the sense of the *hómoion* -principle), however, could then not be rescued any more by assigning them to a natural system of correspondences: for example, the doctrine of signatures. As a reputable physician, one had to avoid being associated with Paracelsus who was in that time of Enlightenment a taboo for scholars. Plain *lógos* -science, on the other hand, also had no place for it either, as qualities in this case are basically reduced to measurement values and processed according to causal-mechanical laws.
3. As a child of his time, Hahnemann was after all also a member of Freemasonry, 49 which suggests ritual-mystic experience of initiation. In addition, through his classical education in the elite high school St. Afra in Meißen and his command of the ancient languages he had (so to say) barrier-free access to the entire Greek and Latin original literature. So, it seems highly improbable that he may not have known the *iásthai* -principle, that the harmful also can heal. Moreover, this idea was then in the air anyway, as is shown by the first vaccination with cowpox by Edward Jenner in 1796, 50 the year of the original publication of Hahnemann's new principle. 51 52

So, Hahnemann had neither just helped modern medicine toward scientific progress nor founded just another simile-therapy in the sense of traditional *hómoion* -thinking, nor just an esoteric elitist healing practice which would be accessible only to few initiates. Rather he was able, on the basis of his idea of systematic drug provings with healthy volunteers,

1. to present his doctrine as scientific in the sense of experimental pharmacology;
2. to connect with simile-thinking, however not directly but mediated by the reaction of the organism to pathogenetic or medicinal stimuli;
3. to do justice to the old healing principle by operationalising it to the concept that a detuning of the vital force, caused by a pathogenetic stimulus, may, nay will, be extinguished by a similar medicinal stimulus. 53 54

## The Roots of Homeopathy

The homeopathy of Hahnemann, thus, has various roots, namely at least the three here distinctively presented traditions of thinking.

1. Homeopathy's claim of scientificity rests on *lógos* -thinking.
2. Its claim of individualisation and saving of the qualities rests on *hómoion* -thinking.
3. Its claim of being an art of healing rests on *iásthai* -thinking.

Contrary to modern iatro-technology which since the alliance of medicine and natural science in the 19th century has committed itself almost exclusively to *lógos* -thinking, homeopathy may be considered as a hybrid of different strands. Composed of heterogeneous and ultimately irreducible parts, it is a fragile entity that by overemphasis, amplification or absolutification of one of its aspects could be damaged. Hence, it is endangered and threatened from several directions.

1. If one tries to understand, prove or disprove homeopathy only on the basis of *lógos* -thinking, as the so-called natural scientific critical school within homeopathy had intended and as advocates of evidence-based medicine keep demanding, the other two dimensions may get lost. One may no longer be able to see that even the most exact clinical studies will always have two weak points or blind spots: on the one hand, the skill (and imponderability) of the therapist to find the simile, and on the other, the restriction of the *iástai* -principle that healing just might succeed in this manner but does not have to.
2. If one tries to practice homeopathy only on the basis of a plain *hómoion* -thinking—that is, establishing simile-analogies of any kind between patient and remedy, from botanical, zoological, astrological, up to psychological and mythological correspondences, as this may be in vogue with a considerable number of modern homeopaths—then also here the other two dimensions may get lost. One may not see that, through this, the scientific claim of homeopathy is abandoned, as well as the core idea of *kátharsis* : that the healing agent not only may superficially match and please, but also must have the capacity to hurt. This, however, may only be detected in remedy provings with healthy humans.
3. If one tries to understand homeopathy only on the basis of a spiritual *iástai* -thinking and is tempted to expand its range of indications to anything one may encounter—for example by ‘proving’ pieces of music, fairy tales or poems and applying these findings therapeutically—then again the other two dimensions may be negated. One may possibly no longer notice that, through this, one's own scientific claim, as well as the substrate of the simile-relation, evaporates.

## The Challenge

Homeopathy, therefore, may always have to meet the enduring challenge to balance and integrate its three constitutional ways of thinking into a vivid concrete and beneficial practice. In addition, socially and politically, it has to find and defend its place and identity within different societies, amidst other competing medical systems. Perhaps, synergies or cooperation might be possible. But how may dissimilar things be reconciled, considering that—according to *hómoion* -thinking—they have the natural tendency to drift apart, as long as there is no extra force to keep them together? Empedokles would say, only under circumstances where not strife but love or friendship ( *philótes* ) prevails.

Once, in the days of Hahnemann, a high degree of love and goodwill was already needed to combine and hold together the different constituents of homeopathy. But at that time—within an atmosphere of general cordial striving for the good—it still seemed to be possible, in the discourse with learned colleagues, such as Hufeland, not to lose sight of the common goal and ethos and to keep an open mind for the truth which was still not exclusively defined by monetary *lógos* -thinking.

## Outlook

Today we seem to live increasingly in an advanced era of collective strife ( *néikos* ), which may be due, not least, to the global hegemony of money-driven *lógos* -thinking. Its inner logic may be appealing, but its social impact could be devastating as it tends to isolate and antagonise individuals. In the wake of industrialisation, capitalisation, commercialisation, digitalisation, in conjunction with materialism, egoism, hedonism and agnosticism, the final evolution of humankind may result in a species called *homo oeconomicus* whose entire ‘world’ has dwindled down to a computable process of rational decision-making.

On the other hand, the principle that similars cling together while dissimilars tend to oppose each other, although tendentiously overlooked by modern medicine and science, is still working in reality, as powerfully as ever. Everybody may observe and verify this, for instance on a socio-political level, in the formation of ever-new ethnic, religious, ideological and national communities and identities, and their corresponding conflicts and wars. Without taking seriously and elevating *hómoion* -thinking from its crude to more sophisticated forms, as Plato and Aristotle did, for example, with an ethical intention when relating it to a common good, idea, or God, modern societies may break apart into ever more homogeneous echo chambers.

Medicine's neglect of *iásthai* -thinking is, finally, just the other side of the coin with regard to how modern health care systems are organised and controlled: that is, on the basis of commodification and commercialisation of medicinal products and technologies, professionalisation and institutionalisation of therapies and strict alliance with reductionist *lógos* -thinking. No wonder that, confined to such a conceptual framework, people feel lost when having to deal with phenomena such as the so-called placebo effect or unexpected cures of allegedly incurable diseases, etc. In modern medical dictionaries the term ‘healing’ is missing anyway.

## Conclusion

Keeping a sound balance between the aforementioned thinking traditions may not only be crucial for homeopathy but also prove to be a persisting task for medicine at large and for societies—if not for the world as a whole. Without a rehabilitation of *hómoion* - and *iásthai* -thinking, the only remaining option for human beings to survive in a merely *lógos* -dominated world may in the end be to transmute into computers or robots themselves.

## Highlights

- Homeopathy seems to be constituted by three powerful traditions of thinking, tracing back to Ancient Greece and pre-Socratic times. They may be termed *lógos* -, *hómoion* - and *iásthai* -thinking, according to their genealogical sources.
- Modern medicine may be characterised as solely relying on the first one, which derives from (and is as such compatible with) the logic of economics predominating the health care systems of modern societies.
- Homeopathy, as being based on a junction of all three distinct ways of thinking, proves to be persistently in need of a vital and dynamic equilibrium of its three constituents.
- Attempts to approach homeopathy from only one of the indicated modes of thinking fail to grasp its essence and result in figments or caricatures of what homeopathy was originally meant to be.
- Apart from being a crucial corrective for guiding homeopathy through the 21st century, the new categories suggested here may also be useful in understanding and handling topical problems of society, politics and culture.

## Notes

**Addendum**

One of the major difficulties and challenges related to this article may well be the imposition of considering the possibility that our usual way of thinking (in terms of *lógos* ) might not be the be-all and end-all of everything, having instead a socio-economic origin in history before which it did not even exist.

There is extensive literature not only on the advent and ascent of monetarism in Western civilisation 1 2 3 but also on the transition from premonetary to monetarised culture and its social, economic, political and philosophical implications, including the constitution of abstract value, the concept of an individual self, the bourgeois subject and its typical form of thinking, up to the peculiar rationality of civic sciences. 4 5 Clearly, without the background knowledge provided there, it may be puzzling for regularly educated modern scientists who are unexpectedly confronted with the thesis that it was not allegedly eternal and consistent scientific reasoning that—amongst others—‘invented’ coinage, but the other way around. Actually, there is no philological or historical evidence of the modern way of thinking in terms of abstraction, modern rationality, etc., in Homeric times or before, neither in Greece nor in the early Near East nor in India, China, etc. It came into being only in Ionia in the 6th century BC, in the wake of the unprecedented spread of coinage. Richard Seaford, on 1200 pages of his three relevant books, passes through (nearly) all possible objections and difficulties unprepared scientists might have in accepting that modern rationality is a product rather than the cause of socioeconomic developments having occurred in Ancient Greece. 6 7 8 To repeat this here may well go beyond the scope of this paper.
Roughly summarised, the leading socioeconomical principles governing pre-Homeric society were honor, prize and exchange of gifts on a personal basis and, especially in Greece, sacrificial communal reciprocity and redistribution, whereas the looming advent of impersonal exchange or trade of commodities, as well as the emergence of cities and temples etc., was seen critically as threatening the traditional aristocratic order of values. Even at the time of Plato, the polis was still mainly held together by communitarian rituals, traditions, religion, etc., rather than by commerce or market trade where individuals tend to make profit at the expense of their partners, thus compromising and undermining their social cohesion. Against this background, it might be understandable that Athens, as an early democratic community, reacted adversely to Anaxagoras' and Socrates' propagation of a new kind of abstract and categorical thinking—that seemed to be appropriate to unholy autocracies but not to the Athenians' cosmos of gods and myths and values.
And yet, this old world of traditional *hómoion* -relations was in decline, at the latest when the sophists publicly offered to teach virtue for money, thus devaluing and liquidating the peculiarity of the dimension of higher aspirations, such as nobleness, virtue, loyalty, personality, character, etc., by asserting its producibility and convertibility by monetary means. The claim of Protagoras that ‘Man be the measure of everything’ is to be seen in that context of reducing the horizon of affirmation to material, physical and wordly desires that may be achieved and equated with a specific amount of money.

Coined pieces of metal can only perform monetary functions if there is confidence in their acceptability within the community, usually secured by sanction of the state as the legal tender. This is a crucial, paradigmatic difference to the previous trade in goods or in kind. Together with the abstraction of monetary value, the introduction of coined money may have facilitated the emergence of a general consciousness or suspicion that this value, or values in general, may not be valuable in themselves or by nature ( *phýsis* ), but just by convention ( *nómos* ). And from this point, by analogy and projection, everything that has been appreciated until then may henceforth have appeared to be human construction, fiction: that is, relative, from qualities of gods to scientific theories. Epistemic relativism, in turn, encouraged individualism in the sense of no longer feeling bound by universal truths, but free to create one's own world view and peculiarity.

Actually, it was Plato's mission to refute the sophists' relativism and materialism, hinting at a postulated difference between truth ( *alétheia* ) and opinion ( *dóxa* ): that is, between a realm of eternal invariable ideas and the region of daily exchange of ephemeral replaceable judgements or feelings. In the 4th century BC Athens was long monetised, and Plato, when trying to explain that reasonability ( *phrónesis* ) is of a different (general) kind than single (concrete) thoughts, could now draw on the analogy of money, where by means of one and the same coin many different things may be exchanged as equivalents. Ultimately, the term and concept of *noús* (reason) also belongs to this sphere of abstract principles that govern and determine everything.

Preceding Plato, Parmenides had already introduced a form of dualism (or graduated monism) of (immutable) ‘being’ ( *ón* ) versus (versatile) ‘non-being’ ( *mé ón* ), probably having in mind the paradigma of circulating money which may also be understood as (1) having a perpetual value which, however, (2) needs to be sustained by permanent circulation on the market. On the level of trade (i.e., circulation), commodities are and remain different, they cannot merge but only mix, but are connected and related by the invisible uniform power of money. Parmenides' metaphysical conception may appear as a perfect analogy to this dialectic.

In Empedokles' philosophy of love and strife, however, the mystic origin seems to be evident. Although his cosmology is already conceptualised in terms of abstract ‘elements’ and an impersonal ‘sphere’, his four elements still bear the names of gods (Zeus, Hera, Nestis and Aidoneus), and their adherents love and enjoy each other. Here, the influence of monetary thinking is still counterbalanced by a strong sense of relatedness in terms of *hómoion* -connections. Well known is, for example, Empedokles' theory of perception, according to which earth is recognised by earth, water by water, fire by fire, etc. 9

This way of thinking and experiencing the world may clearly make for a different (abounding) cosmos, which today may be forgotten or even lost in the common consciousness. Nowadays, the average citizen appears to be socialised to think in terms of *lógos* , not of *hómoion* or *iásthai* . As their major function it seems to be intended to process data and trade with money. For this, *lógos* -thinking suffices. However, as not everybody feels complete and satisfied by this, there is still a chance that thinking along the lines indicated by the terms *hómoion* and *iásthai* may yet be realised.

Hahnemann was not a mere *lógos* -rationalist, but definitely (still) had access to other dimensions of perceiving the world and its ingredients. Thus, for him drug proving was not a neutral academic procedure, but a peculiar personal experience, much wider than what might be expressed by the usual labels of diseases and symptoms. A breath of his attitude may finally be conveyed by a quote from the Organon:

‘Self-provings have … irreplaceable advantages for the physician. First of all, the great truth becomes an undeniable fact for him, that what is medicinal about all medicines (wherein their curative power rests) lies in the condition-alterations undergone by means of the self-proven medicines and in the disease states self-experienced by means of these medicines. … Let him not imagine that such small illnesses from taking proving medicines are generally detrimental to his health. On the contrary, experience teaches that, through the various attacks on the healthy condition, the prover's organism only becomes the more practiced in warding off everything from the external world that is inimical to his body, along with all the artificial and natural disease malignities. By means of such moderate self-provings with medicines, the prover's organism also becomes more seasoned [hardened] against everything that is detrimental. His health becomes more invariable; he becomes more robust, as all experience teaches’. 10 11
